# Supplementary material for: Exploring endothelial cell environments across organs in spatially resolved omics data
Source: bioRxiv. 2025 Sep 25:2025.09.23.678129. Preprint. [Version 1] doi: 10.1101/2025.09.23.678129 (PMC12485928; doi:10.1101/2025.09.23.678129)
Supplement: Supplement 1 [file NIHPP2025.09.23.678129v1-supplement-1.pdf]

bioRxiv preprint doi: https://doi.org/10.1101/2025.09.23.678129; this version posted September 25, 2025. The copyright holder for this preprint (which was not certified by peer review) is the author/funder, who has granted bioRxiv a license to display the preprint in perpetuity. It is made available under aCC-BY 4.0 International license.

Supplementary Tables

Supplementary Table 1. Screenshots of individual EUI organs for studies registered using the RUI.

Organ, Sex	Team	EUI	Anatomical Structures	Notes
Small Intestine, F	Stanford University	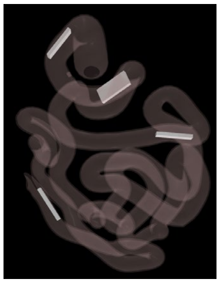	duodenum, proximal jejunum, mid-jejunum, ileum	4 extraction sites
Large Intestine, F	Stanford University	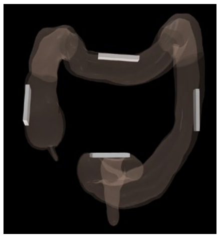	rectum, cecum, appendix, ascending colon, transverse colon, descending colon, sigmoid colon, splenic flexure, hepatic flexure	4 extraction sites
Small Intestine, M	Stanford University	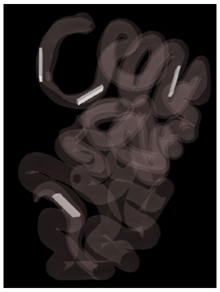	duodenum, jejunum, terminal ileum, ileum	4 extraction sites
Large Intestine, M	Stanford University	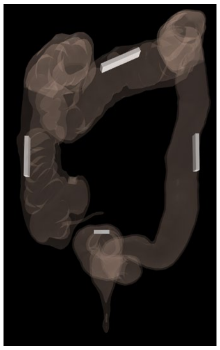	rectum, cecum, ascending colon, transverse colon, descending colon, sigmoid colon, splenic flexure, hepatic flexure	4 extraction sites

bioRxiv preprint doi: https://doi.org/10.1101/2025.09.23.678129; this version posted September 25, 2025. The copyright holder for this preprint (which was not certified by peer review) is the author/funder, who has granted bioRxiv a license to display the preprint in perpetuity. It is made available under aCC-BY 4.0 International license.

Large Intestine, M	Harvard Medical School	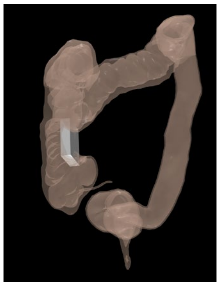	ascending colon	1 extraction site
Lung, F	University of Rochester Medical Center	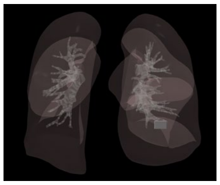	lower lobe of right lung, right posterior basal bronchopulmonary segment	1 extraction site
Lung, M	University of Rochester Medical Center	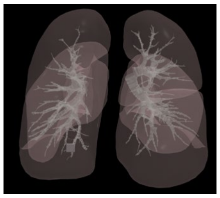	lower lobe of left lung, left posterior basal segmental bronchus	1 extraction site
Lymph node, F	Yale University School of Medicine	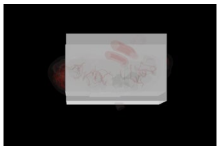	medulla, capsule, mesenteric lymph node	3 extraction sites
Lymph node, M	Yale University School of Medicine	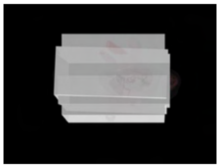	medulla, capsule, mesenteric lymph node	3 extraction sites
Pancreas, F	University of Florida	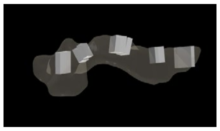	head of pancreas, neck of pancreas, body of pancreas, tail of pancreas	8 extraction sites
Pancreas, M	University of Florida	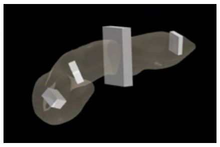	head of pancreas, neck of pancreas, body of pancreas, tail of pancreas	4 extraction sites
Skin, F	General Electric Research	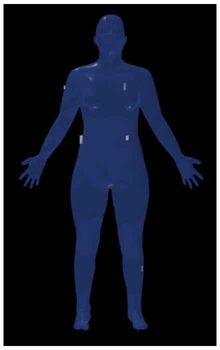	skin of body	6 extraction sites

bioRxiv preprint doi: https://doi.org/10.1101/2025.09.23.678129; this version posted September 25, 2025. The copyright holder for this preprint (which was not certified by peer review) is the author/funder, who has granted bioRxiv a license to display the preprint in perpetuity. It is made available under aCC-BY 4.0 International license.

Skin, M	Harvard Medical School	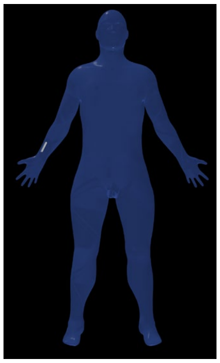	skin of body	1 extraction site
Skin, M	General Electric Research	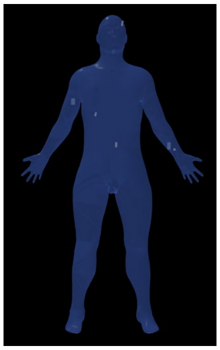	skin of body	6 extraction sites
Spleen, F	Yale University	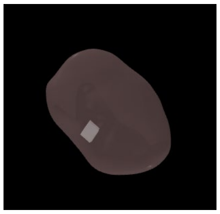	hilum	2 extraction sites
Spleen, M	Yale University	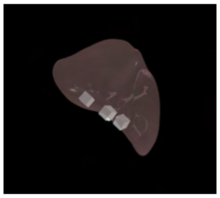	hilum	4 extraction sites
